# Non-Authentic Genotypes—An Unrevealed Problem in Plant Research and Breeding

**DOI:** 10.3390/plants15050788

**Published:** 2026-03-04

**Authors:** Antonín Dreiseitl, Zdeněk Nesvadba

**Affiliations:** 1 Department of Integrated Plant Protection, Agrotest Fyto Ltd., Havlíčkova 2787, CZ-767 01 Kroměříž, Czech Republic; 2 Gene Bank, Czech Agrifood Research Center, Drnovská 507, CZ-161 00 Praha 6, Ruzyně, Czech Republic; zdenek.nesvadba@carc.cz

**Keywords:** cereals, barley, *Hordeum vulgare*, gene banks, powdery mildew, *Blumeria hordei*, resistance gene postulation, mislabeled accession

## Abstract

Genes important for research and breeding plant varieties are crucial for the survival and development of human civilization. Seeds of cereal germplasm are maintained in gene banks (GBs) and grain viability of GB accessions must be regularly restored by seed multiplication. During related operations human errors may lead to contaminated or mislabeled accessions and resultant genotype non-authenticity. Such mistakes accumulate over time. In this report, 1412 lines derived from 289 accessions of 93 barley varieties, each obtained from several GBs, were analyzed. Five single seed progenies (SSPs) were usually harvested from an accession and their major genes conferring powdery mildew resistance were postulated. Twenty-two known resistance genes and 60 of their combinations were identified. Non-authentic genotypes contained different genes compared with genes present in other genotypes of the same variety. Based on these results we found at least 40 (13.8%) mislabeled and 102 (35.3%) heterogeneous accessions in which 276 lines (19.7%) carried non-authentic genotypes. Misrepresented varieties in GBs are a great problem for research projects, especially those focused on finding new (e.g., molecular) varietal characteristics, and in breeding programs as the required gene combination cannot be obtained.

## 1. Introduction

Plants are necessary for maintaining human life and provide nutrition for an increasing population, and authentic germplasm is imperative for executing reliable and repeatable research as well as for breeding new varieties with particular characteristics. Thus, effectiveness of research and breeding depend on the use of true genotypes.

Intraspecific genetic diversity of crops represented by wild resources, landraces and bred varieties is mostly preserved ex situ. Gene banks implement standard procedures to attain accuracy and reliability [1,2], but human mistakes during seed increase and storage are inevitable and accumulate over a period of years. This is particularly so when handling many accessions [3,4,5]. Errors such as confusing identities, duplication or misreading of accessions, all denoted here as mislabeling, result in total non-authenticity of GB accessions and mechanical genotype contamination or outcrossing in their partial non-authenticity. When these are genotyped or sequenced [6,7], faulty genetic data can be linked to mislabeled genotypes, resulting in incorrect or biased results or reduced power to detect real biological patterns [8]. Therefore, control of germplasm identity and genotype purity must be a high priority in research, breeding and conservation activities [9]. However, failure to publicize such discrepancies, especially in cereal GB accessions, means that it is often difficult to recognize aberrant genotypes.

Resistance genes against diseases present in commercial varieties have been usually identified during breeding, trialling or soon after their registration. It is often based on the gene-for-gene model [10,11], which uses pathogen diversity and is designated as gene postulation [12]. It is a classical but still very effective approach that compares the responses of tested varieties with standard host lines containing known resistance gene(s), both inoculated with a set of pathogen isolates. Such genotype characteristics are designated as reaction patterns [13], resistance spectra [14], profiles [15], response type arrays [16] and, in this report, as infection response arrays (IRAs).

Europe is the largest producer of barley (*Hordeum vulgare* L.) and, since powdery mildew caused by *Blumeria hordei*, M. Liu and Hambl., was a predominant disease, breeding resistant varieties was a priority and these possess many major genes of specific resistance and their combinations. An effort to collect information about the genetic basis of varietal resistance led to the identification and publication of the resistance genes in almost 700 European varieties [17]. The primary use of resistance genes is protecting plants against diseases or pests. However, knowledge of these genes in a host has wide utilization in research and breeding. For example, current results of resistance gene identification can be compared with existing original data and on this basis varietal authenticity can be detected and established.

Non-authentic genotypes were a source of complications in our study of powdery mildew resistance genes in winter barley varieties [18], and verification of correct genotypes and detection of non-authentic genotypes became an important topic of our research. When we screened a collection of the wild progenitor of cultivated barley (*H*. *vulgare* subsp. *spontaneum*) lodged in an international GB, a great heterogeneity of accessions was uncovered [19]. The Czech (CZE) GB core collections of bred varieties and landraces of spring and winter barley were subsequently studied [16,20] and when results were compared with older data many non-authentic and heterogeneous accessions were found for reasons outlined above. In a later investigation a smaller set of spring barley varieties originating from seven foreign GBs was pathologically screened and the results compared with those from identically labeled varieties maintained in the domestic GB; 37.5% of the accessions were heterogeneous, and at least 20.0% were mislabeled [21].

The aim of this work was to detect non-authentic genotypes in a set of winter barley varieties originating from numerous GBs. A large number of genotypes was grown, harvested and tested, their powdery mildew resistance genes were postulated, and based on these results non-authentic genotypes were identified.

## 2. Results

### 2.1. Postulation of Resistance Genes

We obtained 196 accessions of 93 winter barley varieties from eight foreign GBs and usually five single seed progenies (SSPs, also called lines or genotypes here) were tested from each (Table S1). In 947 SSPs 12 IRAs indicated the presence of single *Ml* genes (*a6*, *a7*, *a8*, *aLo*, *at*, *g*, *h*, *Ch*, *k2*, *ra*, *Ru2* and *Wo*), nine genes found only in combination with other genes (*a12*, *a13*, *Dr2*, *Dt6*, *He2*, *La*, *Ln*, *Lu* and *VIR*) were characterized only IRAs of standard barley varieties, and 44 IRAs represented combinations of named major genes of specific resistance against powdery mildew and one IRA of susceptibility (absence of specific resistance—*none*).

### 2.2. Collating Previous Results

The results relating to 93 accessions and their 465 SSPs derived from the same varieties of the Czech GB [20] were added to the 947 SSPs tested in this work. In this series an IRA representing *Mla3* and 16 other IRAs established the presence of other combinations of the above-mentioned genes. Thus, in total 83 IRAs characterized all analyzed SSPs and 11 isolates were sufficient to differentiate them (Table S2).

### 2.3. Total Set of Genotypes Analyzed

The results of the postulation studies are presented in Table S1 and relate to 1412 SSPs derived from 289 accessions of 93 varieties obtained from nine GBs and bred or collected in 25 countries of the northern hemisphere. Each variety was represented on average by 3.1 accessions originating from different GBs and by 15.2 SSPs. In 93 varieties 273 genotypes (variety × resistance gene(s)) were identified. The numbers of SSPs and genotypes in an accession and numbers of analyzed accessions and SSPs are presented in Table 1.

### 2.4. Frequency of Postulated Resistance Genes

The frequency of all 22 postulated resistance genes is summarized in Table 2. The most numerous were *MlaLo* found in 368 SSPs, *Mlra* (363) and *Mlh* (278). Most of these were present in combinations with other resistance genes. Genes with the lowest frequency included *Mlk2* detected in three and *Mla3* and *MlHe2*, each present in six SSPs. Unknown (u) resistances were recorded in 97 SSPs, of which 52 were effective against all used pathogen isolates (*uE*) and in contrast 91 SSPs were characterized by the absence of any specific resistance (*none*).

### 2.5. Homogeneous Accessions and Varieties

In 187 accessions no heterogeneity was detected because all 908 SSPs had one identical genotype within an accession and they are considered homogeneous (Table S1). In 25 varieties there was an identical resistance (homogeneous varieties). Of these Zend was represented by nine SSPs derived from two accessions, whereas Sigra by 30 SSPs belonging to six accessions (Table S3).

### 2.6. Heterogeneous Accessions and Varieties

In 102 (35.3%) accessions represented by a total of 504 SSPs, two to five genotypes were found and were, therefore, heterogeneous (Table S1). They belonged to 68 (73.1%) varieties including 248 SSPs and contained from two to eight (average 3.6) genotypes (Table S4).

### 2.7. Mislabeled Accessions

Mislabeled accessions are defined as the presence of different genotype(s) in all or at least four tested SSPs of a given accession compared to the genotype(s) of the other GB accession(s) of the variety. There were 40 (13.8%) mislabeled accessions—34 “different”, at least two “unclassified” and four “non-authentic” (Table S1).

### 2.8. Authentic Genotypes

The known resistances in three SSPs of Nakaizumi-zairai (USA) and five SSPs of Kompolti 4 (CZE) were confirmed. The following can also be considered as authentic, namely 369 SSPs derived from 75 accessions of 25 homogeneous varieties with identical resistances (Table S3), 481 SSPs carrying resistances predominating in the given varieties, 268 assumed authentic lines in 19 heterogeneous varieties (Table S5) and a maximum of 10 unclassified SSPs. Thus, 1136 SSPs represent authentic genotypes (Table S6).

### 2.9. Non-Authentic Genotypes

There were 20 SSPs of confirmed non-authentic accessions in varieties Franger (5), Kompolti 4 (5) and Nakaizumi-zairai (10). Furthermore, 246 SSPs carrying resistance genotypes different from other genotypes of these varieties are likely to be non-authentic as well as at least half of 20 unclassified SSPs. Hence, a minimum of 276 SSPs (19.7%) can be considered as non-authentic (Table S6).

## 3. Discussion

Among the commonest reasons for varietal non-authenticity maintained in GBs are (1) mislabeled accessions, (2) contamination with other genotypes, or (3) reselection and resultant homozygosity of heterogeneous varieties. As there are a large number of genotypes analyzed here, only a few model examples can be highlighted.

Non-authentic accessions resulting from varietal mislabeling. Mislabeling of varieties in GBs can occur during the handling of seeds and it was found in at least 40 out of 289 accessions (Table S1). However, in some cases determining the authenticity of accessions can be difficult to disentangle as in the case of Anson. This variety was obtained from three GBs (Table 3).

Accessions from CZE and USA GBs carried *Mla8*, while the accession from the British GB contained *Mla7*. Thus, accessions from two GBs carrying an identical resistance gene (*Mla8*) could be considered as authentic. However, Anson is a British variety and the accession from its domestic GB had a different resistance. The pedigree of Anson in the Czech GB is Triumph × Yamina, while in the GB of the USA is Volbar × Jefferson. Therefore, they must be different varieties both named Anson and with the same resistance gene. To confuse matters further, Anson obtained from the British and Czech GBs have identical pedigrees but different resistance genes. Therefore, based on resistance genes and pedigree it is not possible to state whether the accessions, their pedigrees or both are incorrect. Similarly, *MlaLo* was detected in 15 SSPs of Slaski II accessions from Czech, German and USA GBs, but in the heterogeneous accession of this Polish variety sent from the Polish GB *MlaLo* was not present.

Four SSPs of the Japanese variety Nakaizumi-zairai from the Czech GB were characterized by the absence of resistance genes (*none*) and one SSP by the presence of *Mla8*, while all SSPs from the German GB contained *MlRu2*. Based on these results it would be impossible to decide which one is authentic. However, this variety is the standard for *Mlk2* [22] and this gene was detected only in all three SSPs derived from the USA accession (Table S1). Thus, both the CZE and DEU accessions cannot be accepted as authentic. A similar example relates to an accession of the Ethiopian spring barley landrace Abyssinian 1102—a known donor of durable Mlo resistance [23]. All homogeneous accessions from GBR and USA collections contained Mlo, while in a heterogeneous CZE accession only lines with *Mla8* and without the resistance gene (*none*) were found. Conversely, in two of five SSPs of the CZE accession of Diamant, and in three of five SSPs of this variety from a Slovakian (SVK) GB, Mlo was detected (CZE and SVK GBs have close cooperation), whereas in five SSPs of Diamant from the British GB the expected *Mla8* allele was present [21].

It was surprising that, although cereal accessions rank among the most numerous in plant GBs, we found no references dealing with mislabeled varieties. Nevertheless, results from GBs of other plant species showed the problem is common and concerning. In yam (*Discorea*), whose tubers are used as a staple food or for natural medicine, 20.6% of the total 3156 GB accessions were not true to type, i.e., misidentified individuals [3]. Af Sätra et al. [24] genotyped apples (*Malus domestica*) and confirmed the identity of multiple accessions with the same variety name but also identified several mislabeled accessions. Shan et al. [25] described the possibility of mislabeled accessions in the world germplasm collections of wild Cicer (chick-pea). Jreisat and Laten [1] reported that 4 of the 13 specimens of clover (*Trifolium*) appear to be mislabeled or misidentified. Van de Wouw et al. [26] found a high degree of non-authentic lettuce (*Lactuca sativa*) varieties in GB collections; this was especially true for the oldest varieties, but even for varieties released from the 1960s to 1990 it was estimated that approximately 10% were not authentic. Zhang et al. [27] applied molecular methods to determine species’ identities in rice (*Oryza*) and revealed that 17% of 53 seed accessions from GBs or field collections were mislabeled. Akpertey et al. [28] analyzed 400 coffee (*Coffea canephora*) genotypes and 18.6% were mislabeled. Parentage analysis showed that 33.3% of the progenies derived from controlled crossing and none of the progenies originating from an open pollinated bi-clonal seed garden had parents (both parents) corresponding to the breeders’ records. Most of results conducted in the plant kingdom and reporting mislabeling of genotypes relate to woody plants such as trees [29,30,31] and shrubs [32,33,34]. Although the situation in GBs of cereals is similar, relevant studies are rare.

Non-authentic genotypes through contamination. The degree of genotypic contamination of accessions can differ and range from just a single seed or plant to the complete replacement of the original variety with another genotype(s). Accession heterogeneity may be welcomed in order to maintain the natural variation within original landraces or varieties bred by crossing that have not undergone line selection. However, in barley GBs the number of modern and almost exclusively single-line varieties has increased and therefore their heterogeneity was not originally present.

Contamination of accessions arises from mechanical admixtures with other genotypes during operations associated with seed multiplication or accidental cross-pollination, both resulting in undesirable heterogeneity. Examples of genotypic contamination in three out of six accessions of three varieties are illustrated in Table 4. In the first case (Freya) five SPPs from the domestic GB possess *Mla6*, whereas two SSPs derived from the DEU GB do not contain this gene. Similarly, nine SSPs derived from two accessions of Virgo carry *MlLa*, *Mlra* but in one SSP a different combination of *Ml* genes was found (a8, h). All these cases (SSPs nos. 2 and 4 of Freya from DEU and no. 2 of Virgo from CZE accession) are considered as random mechanical admixtures (genotype contaminants). Also, one of 10 SSPs of Duet (CZE and GBR) differs from the rest in one of four genes of an unusual gene combination. Therefore, it is probable that this different genotype could have arisen by accidental cross-pollination during seed increase, although a line of the original breeding population cannot be excluded.

A similar case to the above is the heterogeneous variety Bordia (Table 5). Three accessions (DEU, NLD and USA) contained a genotype without a resistance gene (*none*), which could prove the possible authenticity of at least a proportion of seeds of these accessions. Also, some SSPs in the accessions from the USA and GBR could be considered authentic, as *MlaLo* predominated in both. However, if only accessions from German and British GBs were tested, the conclusion would be that these are different varieties as no common genotype was detected in them.

Landjeva et al. [35] studied 91 Bulgarian wheat (*Triticum aestivum* L.) genotypes mostly with microsatellite markers and approximately 74% of the varieties showed heterogeneity. Varshney et al. [36] found heterogeneity in an average of 12.4% of 38 wild barley accessions (*H*. *vulgare* subsp. *spontaneum*) and 5.7% of 185 cultivated genotypes. Ayala et al. [37] analyzed 46 wheat landraces from Andalusia and up to 77 genotypes were found in them. Ertiro et al. [38] studied 265 maize (*Zea mays*) inbred lines from three GBs; only 22% of accessions were considered “pure” with <5% heterogeneity, while the remaining 78% had a heterogeneity ranging from 5.1 to 31.5%. In the germplasm collection of the ornamental crop *Hydrangea macrophylla*, the authors found that 36% of the tested plants were mislabeled [39]. Bairwa et al. [40] analyzed 92 accessions of rice (*Oryza sativa* L.) landrace germplasm with 23 phenotypic traits and 48 microsatellite markers and recorded a large degree of heterogeneity and numbers of heterozygotes in the germplasm.

To uncover heterogeneity depends on the reliability of the method used and may be underestimated here because Dreiseitl [19] found 40.2% heterogeneous wild barley accessions and later more than 85% heterogeneous GB accessions of cultivated winter barley [41] when tested with different numbers of powdery mildew isolates. In this report most of these varieties were tested and only 35.3% of accessions were heterogeneous. This great difference can be explained because in the previous work each accession was represented by about 50 plants, whereas less genotype contamination was uncovered herein since only five SSPs were tested. One might even hypothesize that if an accession of a homogeneous variety of the set is represented with about 100 SSPs and tested with the same set of the pathogen isolates then very few accessions will show full homogeneity, i.e., identical genotypes among all SSPs.

To achieve complete and durable genotype purity of GB accessions of plant varieties is a difficult task while frequent occurrence of non-authentic genotypes in GBs is a significant problem for germplasm users [42]. Therefore, instead of direct use of seed or randomly selected plants from GB accessions it is preferable to prepare and verify authenticity of genotypically pure lines before use in research or breeding. A small number of seeds (10 to 20) should then be made available to ensure reproducibility of experiments [43].

To detect heterogeneity of accessions may be easier than to uncover mislabeled varieties because individual plants or derived lines can be compared concurrently and differences easily seen, whereas accessions incorrectly labeled must be determined using analytical methods similar to those current at the time the variety was bred.

Non-authentic genotypes due to selection. In some GBs accessions can be strictly maintained in their original state, while in others a homogenization of accessions by negative or even positive selection is performed and can lead to gene erosion. Furthermore, a change of curator may modify the approach to maintaining seeds in a GB.

Examples of particular accessions comprising different lines probably through selection are presented in Table 6, which shows resistance genes in nine accessions of two homogeneous varieties. In accessions of Frost from GBs CZE and DEU, a combination of two *Ml* genes (*a6*, *h*) was postulated, while in accessions from GBs SVK and SWE one more gene (*a6*, *h*, *VIR*) was found. This suggests that the original Frost was composed of two lines and progenies of both are maintained in different GBs.

Similarly, the accession Protidor (DEU) was characterized by *Mla12*, *MlVIR*, while the FRA, GBR and USA accessions and three of the five SSPs of the CZE accession additionally contained *Mlg*. In two other SSPs of the CZE accession only *Mlg*, i.e., the gene that was absent in the DEU accession, was detected. It is, therefore, likely that Protidor was originally formed by several lines carrying different resistance genes and their combinations, and different genotypes of this variety are maintained in GBs.

Genetically pure lines can be better characterized than heterogeneous accessions. However, selection results in loss of genes, reduced genetic diversity and increased genetic erosion [44]. Since each gene is linked with other genes, then even selections within a variety might contain or lack powdery mildew resistance genes as well as other alleles that determine different traits. Therefore, if a variety was originally heterogeneous and a pure line was selected for the GB, then such an accession might have lost some of its original characteristics and authenticity.

We can conclude that use of resistance genes against diseases such as mildews and rusts in cereals is a very powerful and precise way for verifying the identity of accessions. Despite that, this method, as for most methods generally, has some limits and detection of non-authentic accessions can be underestimated. Here four groups of accessions carrying exclusively or predominantly genotypes with single *Ml* resistance genes *a8*, *aLo* and *Ch*, and also *none* (no resistance gene), were most frequent. If, for example, Cenad 450 and Oksamyt (both have *MlaLo*) are mixed together, then it is not possible to detect non-authenticity of these varieties using the resistance gene postulation method.

The underestimation of non-authentic accessions can also be partly attributed to the fact that genotypes which were more frequent were usually considered as authentic. For example, the 10 predominant genotypically identical lines in Anson were thought to be authentic whereas three lines from its domestic GB were designated as non-authentic. However, the converse interpretation would indicate that the number of non-authentic genotypes would be higher. Similarly, heterogeneity of accessions may be higher because we studied a maximum of only five lines (SSPs) from each accession and accessions with fewer different components may not have been detected.

Human activities including those in plant GBs are subject to errors resulting in confusion of items. Despite the excellent systems of reducing such mistakes in human medicine there are similar occasional errors in medical data [45], vaccines [46], blood accessions [47] and patients’ identities, organs or body parts [48] and inadvertent exchanges of eggs and sperm [49], embryos [50] and even born children [51]. Thus, human mistakes cannot be avoided even in well-secured systems and GBs are no exception.

In this study we found a high proportion of non-authentic genotypes in barley GB accessions. This finding confirms our previous research as well as results of studies of GB accessions of some other plants. Mislabeled accessions or randomly selected incorrect genotypes from heterogeneous accessions result in the faulty characterization of varieties which can drastically devalue the results of subsequent research and cannot efficiently combine the required traits in breeding.

The problem with non-authentic accessions is general and GB staff must be scrupulous and consistently follow updated methodological procedures [52] for maintaining authentic varieties. In the effort to reduce the number of non-authentic accessions, national gene banks could act as guarantors of the authenticity of their domestic varieties. To this end, they could request respective accessions from other gene banks and use standard varietal characteristics, appropriate methods [53,54] and available information to review their authenticity. No samples should be kept in only one gene bank, but conversely, no samples should be held in multiple gene banks. Nevertheless, it is unreasonable to expect complete authenticity of GB accession at least for the foreseeable future. Therefore, the responsibility for using authentic genotypes in research and breeding projects rests with scientists and breeders. Before doing experiments, they should obtain accessions from different sources (GBs) and verify the required varietal attributes with suitable methods.

## 4. Materials and Methods

### 4.1. Plant Material

Previously, 172 varieties, forming the Czech (CZE) winter barley core collection, were studied [20]. For the current research varieties that were mainly heterogeneous and varieties with some doubt about their authenticity were requested from other GBs. Eight foreign GBs: German (DEU), French (FRA), British (GBR), Dutch (NLD), Polish (POL), Slovak (SVK), Swedish (SWE) and American (USA) responded positively, sending a total of 196 accessions of 93 varieties. Each variety from a given GB was represented by one accession except Stupicky Sestirady—a Czech landrace for which four accessions were received from the USA GB. The full designation of the accessions including their variety names, country of origin and accession numbers is listed in Table S1 and affiliation of the nine gene banks (including domestic CZE) is summarized in Table S7.

The accessions were individually sown in the field in 50 m plots with rows 1 m long and 0.2 m between them, without irrigation, but using chemical weed control in autumn and spring to obtain five SSPs from each. The full number of SSPs was achieved for 177 accessions, while in 19 accessions the number of harvested SSPs was lower due to poor seed germination (Table S8). Thus, instead of the expected 980 SSPs, 947 were tested. To these were added the results from 465 SSPs that had been previously studied and which represented 93 identically labeled varieties from the CZE GB [20]. Thus, a total of 1412 SSPs derived from 289 accessions were analyzed (Table S7). The numbers of varieties and their accessions originating from the various GBs are summarized in Table S9 and the number of SSPs derived from an accession and the total numbers of analyzed accessions and SSPs are presented in Table S10.

### 4.2. Pathogen Isolates

For resistance tests, 52 reference isolates were selected according to virulence/avirulence combinations and obtained from the gene bank of the pathogen maintained at the Department of Integrated Plant Protection, Kroměříž. These isolates had been collected in 11 countries in all six non-polar continents over a period of 66 years (1953–2018) and comprised the global virulence/avirulence diversity of the pathogen. Before inoculation, isolates were checked for their purity and their correct pathogenicity phenotypes were verified on standard barley lines [55]. The isolates were multiplied on leaf segments of the susceptible variety Bowman.

### 4.3. Testing Procedure

Approximately 20 seeds of each SSP were sown in a pot (80 mm diameter) filled with a gardening peat substrate and placed in a mildew-proof greenhouse under natural daylight with temperatures 15 ± 10 °C and relative humidity of 60–90%. The primary leaves were excised when the second leaves were emerging, and leaf segments of about 15 mm long were cut from the middle part of healthy fully expanded leaves. One leaf segment of each SSP was placed on the surface of the media (0.8% water agar containing 40 mg^−L^ of benzimidazole—a leaf senescence inhibitor) in a 150 mm Petri dish. Leaf segments were placed adjacent to each other along with four segments of susceptible check cv. Bowman oriented diagonally in a dish with adaxial surfaces facing upward.

For inoculation, a cylindrical metal settling tower of 150 mm diameter and 415 mm in height closed at the top was used, and a dish with segments was placed at the bottom of the tower. Conidia of each isolate taken from a leaf segment of the susceptible variety with fully developed pathogen colonies were shaken onto a piece of black paper to visually control the amount of inoculum deposited. Then, the paper was rolled to form a blowpipe and conidia of the isolate were blown into the settling tower over the Petri dish at a concentration of ca. 10 conidia mm^−2^. The dishes with inoculated leaf segments were incubated at 20 ± 1 °C under cool-white fluorescent lamps providing 12 h light at 30 ± 5 μmol·m^−2^·s^−1^.

### 4.4. Evaluation

**Figure 1:**
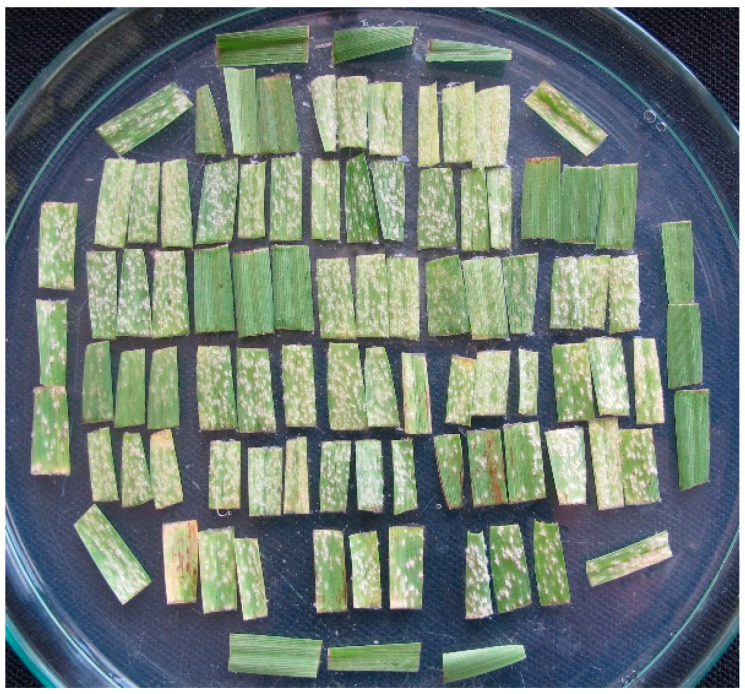
Petri dish with 90 leaf segments of tested barley genotypes and four diagonally placed segments of a susceptible check variety seven days after inoculation with a powdery mildew isolate.

First scoring of infection responses (IR = phenotype of a host SSP–pathogen isolate interaction) was done seven days after inoculation on a scale of 0–4, where 0 = no mycelium, sporulation, necrosis or chlorosis, and 4 = strong mycelial growth and sporulation, no necrosis or chlorosis [14]; IRs 3, 3–4 and 4 were considered susceptible (Figure 1). Second scoring was done a day later and on the same day differences were re-scored. In case of unclear IRs or doubts about postulated resistances additional tests were done. During phenotyping, special attention was paid to boundary IRs 2–3 and 3, which pose the greatest risk of error in distinguishing between resistance and susceptibility [56]. Based on the gene-for-gene model, the resistance genes in SSPs were postulated by comparing their IRAs with earlier determined IRAs of standard barley genotypes possessing known resistance genes. Details of the host resistance gene postulation method, which is similar to that used for defining the virulence of pathogen isolates, have been recently described and demonstration images shown [57].

### 4.5. Classifying the SSPs

Basic classification of SSPs of tested varieties was on the basis of authenticity and non-authenticity, but to proceed efficiently more categories had to be applied (Table S6). Identical genotypes present in all SSPs of a variety, genotypes predominating in a variety and also lines that carried common genes of a variety were classified as authentic. Non-authentic genotypes carried different genes compared with genes present in other genotypes of the same variety.

## Figures and Tables

**Table 1:** The numbers of single seed progenies (SSPs) and genotypes in an accession of winter barley, and numbers of analyzed accessions and SSPs.

No. of SSPs in an Accession	No. of Genotypes in an Accession	No. of Analyzed Accessions	No. of Analyzed SSPs
*Homogeneous accessions*			
2	1	3	6
3	1	6	18
4	1	6	24
5	1	172	860
Sum		187	908
*Heterogeneous accessions*			
3	2	2	6
4	2	2	8
5	2	61	305
5	3	25	125
5	4	10	50
5	5	2	10
Sum		102	504
Total		289	1412

**Table 2:** Frequency of 22 *Ml* powdery mildew resistance genes found in 1412 single seed progenies derived from 289 accessions of 93 winter barley varieties.

*Ml* Resistance	Frequency of Resistance Genes		
Gene	In Accessions Tested Herein	In Previously Tested Accessions ^1^	Total
*a3*	0	6	6
*a6*	80	28	108
*a7*	40	10	50
*a8*	151	82	233
*a12*	21	4	25
*a13*	26	11	37
*aLo*	248	120	368
*at*	10	0	10
*Dr2*	87	51	138
*Dt6*	4	5	9
*g*	43	23	66
*h*	194	84	278
*He2*	5	1	6
*Ch*	134	79	213
*k2*	3	0	3
*La*	5	4	9
*Ln*	5	6	11
*Lu*	129	75	204
*ra*	235	128	363
*Ru2*	95	59	154
*VIR*	44	15	59
*Wo*	8	5	13
*u*	35	10	45
*uE*	36	16	52
Sum	1638	822	2460
*none*	59	32	91
Total	1697	854	2551

^1^ Dreiseitl and Nesvadba [20].

**Table 3:** Mislabeled gene bank accessions of three winter barley varieties determined by the presence of *Ml* powdery mildew resistance genes.

Variety	Order No. of SSP ^1^	Origin of Gene Bank Accession(s) ^2^	*Ml* Gene	Origin of Gene Bank Accession	*Ml* Gene(s)
Anson	1	CZE, USA	*a8*	GBR	*a7*, *h*
Anson	2	CZE, USA	*a8*	GBR	*a7*
Anson	3	CZE, USA	*a8*	GBR	*a7*
Anson	4	CZE, USA	*a8*		
Anson	5	CZE, USA	*a8*		
Nakaizumi-zairai	1	CZE	*none*	DEU	*Ru2*
Nakaizumi-zairai	2	CZE	*none*	DEU	*Ru2*
Nakaizumi-zairai	3	CZE	*none*	DEU	*Ru2*
Nakaizumi-zairai	4	CZE	*none*	DEU	*Ru2*
Nakaizumi-zairai	5	CZE	*a8*	DEU	*Ru2*
Slaski II	1	CZE, DEU, USA	*aLo*	POL	*a8*
Slaski II	2	CZE, DEU, USA	*aLo*	POL	*Ch*, *ra*
Slaski II	3	CZE, DEU, USA	*aLo*	POL	*Ch*, *ra*
Slaski II	4	CZE, DEU, USA	*aLo*	POL	*a8*
Slaski II	5	CZE, DEU, USA	*aLo*	POL	*a8*

^1^ SSP—single seed progeny. ^2^ CZE—Czech Republic, DEU—Germany, GBR—Great Britain, POL—Poland, USA—United States of America.

**Table 4:** Genotype heterogeneity of three winter barley varieties in different gene bank accessions determined by the presence of *Ml* powdery mildew resistance genes in derived single seed progenies (SSPs).

Variety	Order No. of SSP	Origin of Gene Bank Accession ^1^	*Ml* Gene(s)	Origin of Gene Bank Accession ^1^	*Ml* Gene(s)
Freya	1	CZE	*a6*	DEU	*a6*
Freya	2	CZE	*a6*	DEU	*none*
Freya	3	CZE	*a6*	DEU	*a6*
Freya	4	CZE	*a6*	DEU	*none*
Freya	5	CZE	*a6*	DEU	*a6*
Virgo	1	CZE	*La*, *ra*	SVK	*La*, *ra*
Virgo	2	CZE	*a8*, *h*	SVK	*La*, *ra*
Virgo	3	CZE	*La*, *ra*	SVK	*La*, *ra*
Virgo	4	CZE	*La*, *ra*	SVK	*La*, *ra*
Virgo	5	CZE	*La*, *ra*	SVK	*La*, *ra*
Duet	1	CZE	*a6*, *Dt6*, *g*, *h*	GBR	*a6*, *Dt6*, *g*, *h*
Duet	2	CZE	*a6*, *Dt6*, *g*, *h*	GBR	*a13*, *g*, *h*, *u(Dt6?)*
Duet	3	CZE	*a6*, *Dt6*, *g*, *h*	GBR	*a6*, *Dt6*, *g*, *h*
Duet	4	CZE	*a6*, *Dt6*, *g*, *h*	GBR	*a6*, *Dt6*, *g*, *h*
Duet	5	CZE	*a6*, *Dt6*, *g*, *h*	GBR	*a6*, *Dt6*, *g*, *h*

^1^ CZE—Czech Republic, DEU—Germany, GBR—Great Britain, NLD—Netherlands, SVK—Slovakia, USA—United States of America.

**Table 5:** Genotype differences of winter barley variety Bordia in four gene bank accessions determined by the presence of *Ml* powdery mildew resistance genes in derived single seed progenies (SSPs).

Variety	Order No. of SSP	Origin of Gene Bank Accession ^1^	*Ml* Gene	Origin of Gene Bank Accession ^1^	*Ml* Gene
Bordia	1	DEU	*none*	NLD	*Ch*
Bordia	2	DEU	*none*	NLD	*none*
Bordia	3	DEU	*none*	NLD	*none*
Bordia	4	DEU	*none*	NLD	*none*
Bordia	5	DEU	*none*	NLD	*none*
Bordia	1	USA	*none*	GBR	*aLo*
Bordia	2	USA	*aLo*	GBR	*aLo*
Bordia	3	USA	*aLo*	GBR	*Ch*
Bordia	4	USA	*aLo*	GBR	*Ch*
Bordia	5	USA	*aLo*	GBR	*aLo*

^1^ DEU—Germany, GBR—Great Britain, NLD—Netherlands, USA—United States of America.

**Table 6:** Different combinations of *Ml* powdery mildew resistance genes found in single seed progenies (SSPs) derived from gene bank accessions of two winter barley varieties—a case of selection.

Variety	Order No. of SSP	Origin of Gene Bank Accession(s) ^1^	*Ml* Genes	Origin of Gene Bank Accessions	*Ml* Genes
Frost	1	CZE, DEU	*a6*, *h*	SVK, SWE	*a6*, *h*, *VIR*
Frost	2	CZE, DEU	*a6*, *h*	SVK, SWE	*a6*, *h*, *VIR*
Frost	3	CZE, DEU	*a6*, *h*	SVK, SWE	*a6*, *h*, *VIR*
Frost	4	CZE, DEU	*a6*, *h*	SVK, SWE	*a6*, *h*, *VIR*
Frost	5	CZE, DEU	*a6*, *h*	SVK, SWE	*a6*, *h*, *VIR*
Protidor	1	DEU	*a12*, *VIR*	FRA, GBR, USA, CZE	*a12*, *g*, *VIR*
Protidor	2	DEU	*a12*, *VIR*	FRA, GBR, USA, CZE	*a12*, *g*, *VIR*
Protidor	3	DEU	*a12*, *VIR*	FRA, GBR, USA	*a12*, *g*, *VIR*
Protidor	4	DEU	*a12*, *VIR*	FRA, GBR, USA, CZE	*a12*, *g*, *VIR*
Protidor	5	DEU	*a12*, *VIR*	FRA, GBR, USA	*a12*, *g*, *VIR*

^1^ CZE—Czech Republic, DEU—Germany, FRA—France, GBR—Great Britain, SVK—Slovakia, SWE—Sweden, USA—United States of America.

## Data Availability

All final data generated or analyzed during this study are included in this article and its supplementary tables. The original raw phenotypic data are not publicly available because of frequent inconsistencies once the first obtained data (IRAs) were subsequently corrected by the results of additional tests, and also owing to the incompleteness of additional tests, when only selected pathotypes were sufficient to confirm or refute the hypothesis about the resistance genes of the tested genotypes. Original raw phenotypic data are available from the corresponding author on reasonable request.

## Supplementary Materials

The following supporting information can be downloaded at: https://www.mdpi.com/article/10.3390/plants15050788/s1. Table S1: One thousand four hundred and twelve single seed progenies (SSPs) derived from 289 accessions of 93 winter barley varieties obtained from nine gene banks, their country of origin, accession number, postulated *Ml* resistance gene(s) against powdery mildew and classification of varietal genotype; Table S2: Eighty-three infection response arrays (IRAs) produced after inoculation with 11 *Blumeria hordei* (*Bh*) isolates on 1412 single seed progenies derived from 289 gene bank accessions of 93 winter barley varieties and the corresponding *Ml* powdery mildew resistance genes; Table S3: Twenty-five homogeneous varieties of winter barley and numbers of their tested single seed progenies (SSPs) carrying identical resistance genes against powdery mildew; Table S4: Sixty-eight heterogeneous varieties of winter barley and numbers of their genotypes; Table S5: Numbers of authentic and non-authentic single seed progenies (SSPs) found in 19 heterogeneous varieties of winter barley; Table S6: Numbers of single seed progenies (SSPs) derived from 289 accessions of 93 winter barley varieties obtained from nine gene banks and classified according to their postulated resistance genes against powdery mildew; Table S7: Nine gene banks involved in the study and the numbers of provided winter barley accessions and derived single seed progenies (SSPs); Table S8: Nineteen winter barley accessions of which fewer than five single seed progenies (SSPs) were tested; Table S9: Numbers of winter barley varieties and their accessions originating from various gene banks; Table S10: Numbers of single seed progenies (SSPs) derived from a winter barley accession and total number of analyzed accessions and SSPs.

## Abbreviations

The following abbreviations are used in this manuscript:

GB	Gene bank
IR	Infection response
IRA	Infection response array
SSP	Single seed progeny
